# The role of anti-eosinophilic therapies in eosinophilic granulomatosis with polyangiitis: a systematic review

**DOI:** 10.1007/s00296-023-05326-1

**Published:** 2023-04-21

**Authors:** Ioannis Kouverianos, Andreas Angelopoulos, Dimitrios Daoussis

**Affiliations:** 1 Medical Graduate, University of Patras Medical School, Patras, Greece; 2 Internal Medicine Resident, General Hospital of East Achaia, Kalavryta, Greece; 3 Department of Rheumatology, Patras University Hospital, University of Patras Medical School, Patras, Greece

**Keywords:** Eosinophilic granulomatosis with polyangiitis, Egpa, Mepolizumab, Benralizumab, Anti-neutrophil cytoplasmic antibodies, ANCA

## Abstract

Eosinophilic granulomatosis with polyangiitis (EGPA) is an antineutrophil cytoplasmic antibody (ANCA)-associated vasculitis, mostly affecting small-sized arteries and usually occurring in patients with an allergic background. Eosinophils seem to play a significant role in the pathogenesis of the disease and, therefore, biologics targeting interleukin 5 (IL5), a cytokine tightly linked to eosinophils, have emerged as a promising therapeutic tool. A systematic review of Medline was conducted from 2007 to 2022 to search for data regarding the use of anti-IL5 therapies in patients with EGPA. Ongoing or unpublished trials were also searched in ClinicalTrials.gov and the World Health Organization trials portal. The efficacy and safety of mepolizumab, an anti-IL5 monoclonal antibody (mAb), was confirmed by a randomized controlled trial (RCT), although a significant proportion of patients did not respond to this treatment. Other studies showed that both doses of 100 mg and 300 mg of mepolizumab are almost equally effective in EGPA. Benralizumab, an anti-IL5 receptor mAb has preliminary promising results and an RCT is planned to be conducted. Apart from their clinical efficacy in EGPA, anti-IL5 therapies may have steroid-sparing properties. Anti-IL5 therapies seem to be effective and safe in patients with refractory/relapsing EGPA and can be used as a steroid-sparing treatment. Nevertheless, more research is needed to clarify the pathophysiology of the disease; this may potentially lead to the identification of biomarkers to pinpoint patients most likely to respond to anti-IL5-blockade.

## Introduction

Eosinophilic granulomatosis with polyangiitis (EGPA), previously known as Churg-Strauss syndrome, is a systemic vasculitis classified within Anti-Neutrophil Cytoplasmatic Antibody (ANCA)-associated vasculitides, alongside granulomatosis with polyangiitis and microscopic polyangiitis [1]. EGPA mainly affects the small-sized arteries leading to clinical manifestations from many organs, such as lungs, skin, nervous system, kidneys, gastrointestinal and cardiovascular systems. The pathogenesis of the disease is not yet clear, but like many other autoimmune diseases there is strong evidence of environmental and genetic involvement [2]. Although it is an ANCA vasculitis, there is strong evidence that eosinophils play a crucial role in the development of the disease. All patients with EGPA have an allergic background; asthma and/or allergic sinusitis appear in 96–100% of them, with the respiratory system being the most affected system by EGPA [3, 4]. Eosinophilic inflammation is one of the cardinal features in EGPA [2]. The cornerstone therapy of EGPA includes oral corticosteroids and other immunosuppressive agents such as cyclophosphamide, rituximab, mycophenolate, azathioprine and methotrexate. Taking into account that IL5 is the master cytokine controlling eosinophil function [3], mAbs against IL5 or IL5 Receptor, such as mepolizumab and benralizumab, respectively, have emerged as promising therapeutic tools in patients with relapsing/refractory disease [3, 5–7]. Mepolizumab has proven efficacy and is included in the 2021 Guidelines of the American College of Rheumatology/Vasculitis Foundation as a treatment option for relapsing EGPA [8]. In this systematic review we focus on exploring the efficacy and safety of IL5 therapies in patients with EGPA. Moreover, we provide a short overview of eosinophil function as well as their role in EGPA pathogenesis.

- Overview of eosinophil physiology

Eosinophils are white blood cells originating from the bone marrow [9] and the average total number in the bloodstream is normally < 500 mm^3^ cells. Eosinophilia is defined as eosinophil cell count > 500 mm^3^ and hypereosinophilia > 1500 mm^3^ [9]. Eosinophils are engaged in a broad spectrum of processes from battling parasitic, bacterial, viral infections and taking part in allergic reactions to hypereosinophilic syndromes and certain types of cancer [9, 10]. In the bone marrow granulocytes, under the effect of specific cytokines such as IL5, IL3 and Granulocyte–macrophage colony-stimulating factor (GM-CSF), differentiate into eosinophils and basophils [11]. Eosinophils are activated by IL5 to proliferate, migrate to the damaged tissue and enhance the inflammation process. IL5 is mostly produced by Th2 cells, Innate Lymphoid Cells-2 (ILC-2), CD34^+^ progenitor cells, Natural Killer (NK) cells, mast cells as well as the eosinophil itself in an auto/paracrine manner. Eosinophils possess IL5 receptors and IL5 can bind to and activate them. Chemokines, especially CCL11, CCL24 and CCL26, play a crucial role in the migration and recruitment of eosinophils in the inflamed tissue/organ. These chemokines are attached to CCR3 receptors on eosinophils and as a result eosinophils are transferred to the damaged tissue [7]. Eosinophils have the ability to phagocyte, secrete harmful proteins such as major basic protein (MBP), eosinophil peroxidase (EPO), eosinophil-derived neurotoxin (EDN) and also cytokines such as IL4, IL5, IL13, IL25 to activate the immune system and maintain inflammation [2].

- Pathogenesis of EGPA. The role of eosinophils

The main histological feature in EGPA is a necrotizing vasculitis with extravascular granulomas that occur mostly in patients with asthma and tissue eosinophilia [2, 12]. In EGPA, granulomas consist of aggregated macrophages and eosinophils in the damaged tissue, mainly locating in the upper/or lower respiratory tract [5, 13]. According to the prevailing pathogenetic model, circulating unprimed neutrophils are activated and attracted to the inflamed region by alternative complement pathway and proinflammatory cytokines such as TNFα and IL1β. Primed neutrophils, under the effect of ANCAs, excrete Reactive Oxygen Species (ROS) and proteolytic enzymes [5]. Furthermore, IL5-activated eosinophils degranulate harmful enzymes which cause endothelium damage; it is a well-known fact that neutrophils produce neutrophil extracellular traps (NETs), but it was only recently discovered that eosinophils are also able to form extracellular traps, contributing to inflammation and thrombogenesis [11]. Increased number of eosinophils is a hallmark of EGPA; the disease is strongly associated with IL5, which promotes the maturation of eosinophils. Patients with EGPA have high serum levels of IL5; on the other hand, IL10, which is an anti-inflammatory and immunoregulatory cytokine, is decreased [14, 15].

**Fig. 1:**
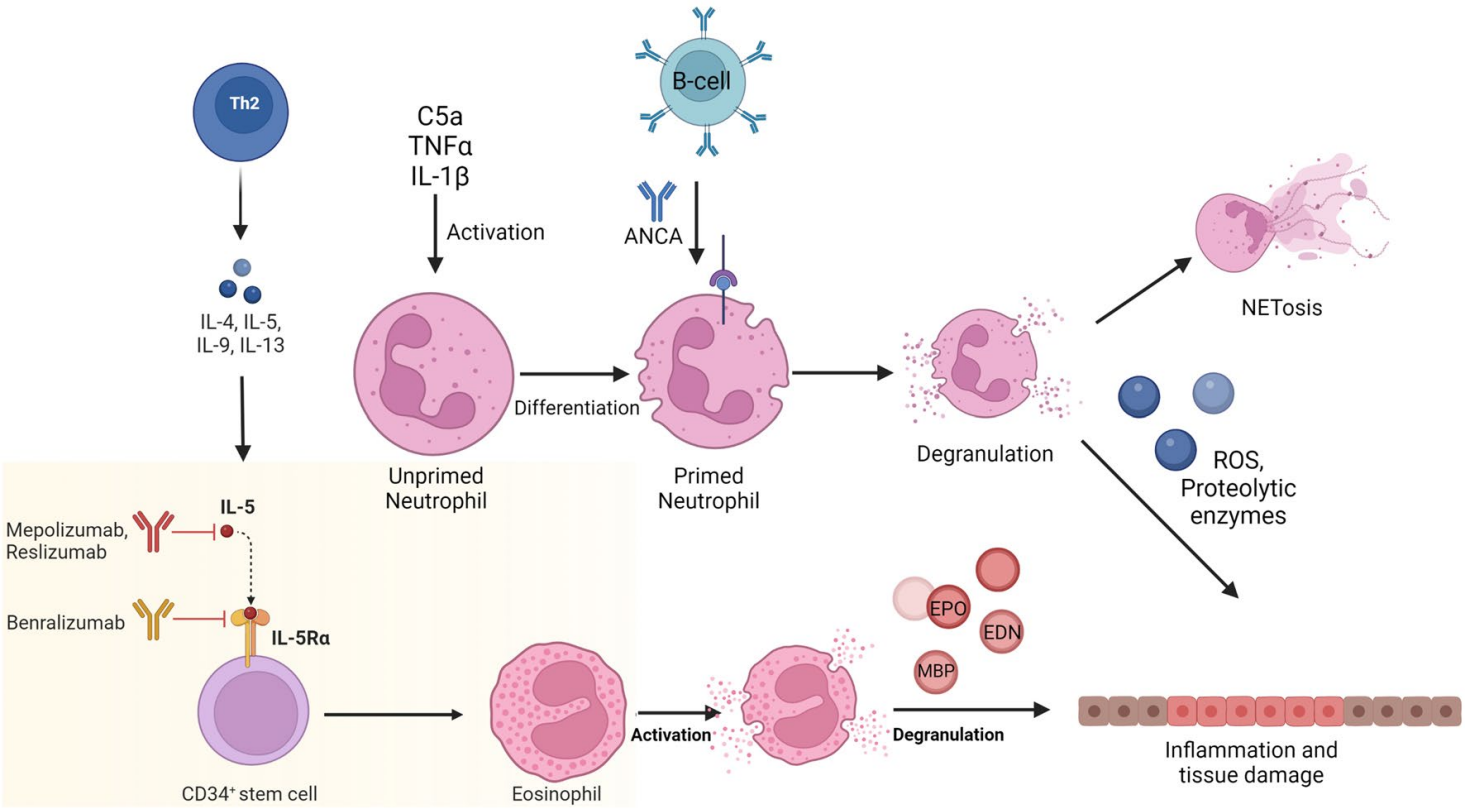
The main pathogenetic pathways in EGPA. ANCA-mediated vascular inflammation and eosinophilic driven pathology

Patients with EGPA can be genetically distinguished into 2 groups; the first group characterized by HLA-DQA1 polymorphisms has positive MPO-ANCAs, whereas the second group has no association with HLA-DQA1 and is MPO-ANCA negative [16, 17]. Although EGPA is classified as an ANCA-associated vasculitis, the percentage of patients with ANCA (mostly MPO/p-ANCA) varies among several studies and fluctuates between 30 and 60% [7, 16, 18]. Nevertheless, all patients, regardless of their ANCA status, have eosinophilia and allergic background. The majority of patients, irrespective of ANCA status, are diagnosed with ear/nose/throat manifestations and lung involvement. ANCA-positive patients frequently have peripheral neuropathy and kidney involvement, in contrast to ANCA-negative patients who mainly have pulmonary infiltrates and may develop cardiovascular involvement [7, 19–22]. Response rates with rituximab in EGPA are significantly higher in ANCA ( +) patients compared to ANCA (−) patients (80% versus 38%, respectively). On the other hand, anti-eosinophilic therapies, such as mepolizumab, may have better response rates in ANCA (−) patients [21, 23]. Figure 1 depicts the current pathogenetic model of EGPA and highlights the role of eosinophils.

## Methods

**Fig. 2:**
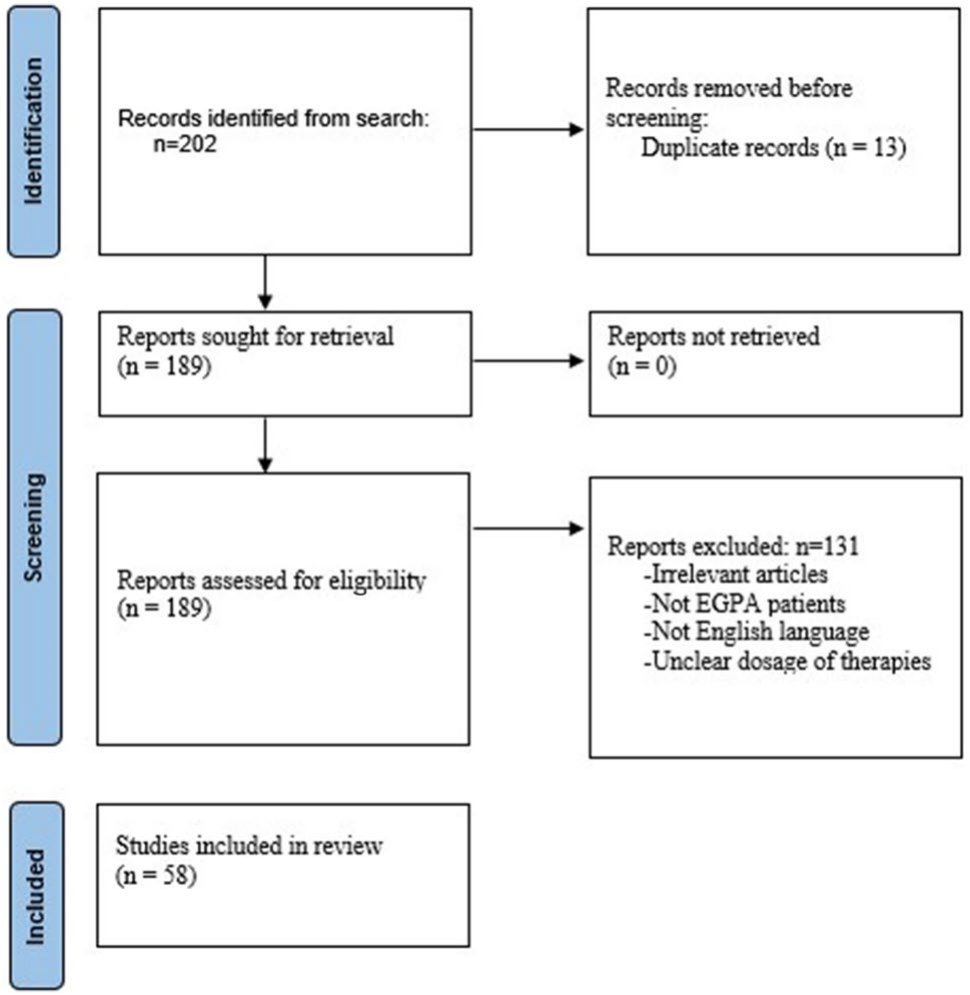
Flowchart of a search strategy

We performed an electronic search on Medline using the keywords: EGPA, mepolizumab, benralizumab, IL5 and ANCA status in the following combinations: EGPA mepolizumab (*n* = 115), EGPA benralizumab (*n* = 32), EGPA anti-IL5 (*n* = 22), EGPA ANCA status (*n* = 33) and limited our search to papers published during the last 15 years. Our research was carried out from September 2022 to December 2022. Ongoing or unpublished trials were also searched in ClinicalTrials.gov and the World Health Organization trials portal. In total, 202 articles were retrieved. We excluded 13 duplicate records and finally, 189 articles were screened. Our inclusion criteria were: (i) studies in the English language and (ii) studies reporting data of anti-IL5 therapies in humans with confirmed EGPA. Finally, 58 articles were included in the analysis. A detailed flowchart depicting the methodology used is shown in Fig. 2.

## Results

### Anti-IL5 therapies in EGPA

#### Mepolizumab

The first clinical evidence of the successful use of mepolizumab in EGPA was reported in 2010 in a case report [24]. However, the efficacy of anti-IL5 agents in EGPA was strongly supported by data derived from an RCT in 2017. This double-blind, multicenter, phase 3 trial recruited patients with relapsing or refractory EGPA who were treated for at least 4 weeks with a standard of care and aimed to evaluate the efficacy and safety of mepolizumab compared to placebo. In this trial-(MIRRA trial) 136 patients were recruited and divided randomly into two groups; 68 patients were treated with mepolizumab, and the other 68 patients received placebo. All patients had a history of asthma associated with eosinophilia and most of them had a history of abnormalities in the sinonasal cavities. The first group of patients started mepolizumab 300 mg subcutaneously every 4 weeks in addition to steroids for a period of 52 weeks. Patients treated with mepolizumab experienced a longer time of remission compared to patients in the placebo group (28% versus 3%). At weeks 36 and 48 the remission rate in the mepolizumab group was 32%, compared to only 3% in the placebo group. The steroid doses in patients treated with mepolizumab were lower compared to the placebo group (44% of the mepolizumab group were able to taper the prednisone dose to 4 mg or less per day, as compared with 7% receiving placebo).

No major safety signals occurred. The most common adverse effect in the mepolizumab group was headache followed by arthralgia, sinusitis, upper respiratory tract infection (URI) and nasopharyngitis. The least common but most severe adverse event was the worsening of asthma.

It is noteworthy that a total of 47% of the patients in the mepolizumab group did not experience remission during the trial. It is unknown why these patients did not respond to mepolizumab. This fact could be explained by the heterogenous profile of EGPA which means that probably a subgroup of patients did not have an eosinophil-associated disease but mostly had vasculitic manifestations not responding to anti-eosinophilic therapies [23]. Moreover, another consideration is that mepolizumab may not be able to infiltrate inflamed tissues to inhibit and deplete eosinophils, although it can reduce the total number of eosinophils in the bloodstream; it is well known that eosinophils may survive due to other cytokines, except IL5, such as GM-CSF [9].

In conclusion, this study showed that mepolizumab was effective and safe in patients with refractory/relapsing EGPA as an additional therapy on top of steroids and has a significant steroid-sparing effect. However, we should underline that almost half of patients treated with mepolizumab did not respond and, therefore, further research is needed [23]. This study included less than 10% ANCA-positive patients and therefore conclusions regarding treatment response according to ANCA status cannot be drawn [23].

Condreay et al. explored if there is an association between polymorphisms associated with the disease and response to mepolizumab therapy. They sequenced 13 genes (IL5, AKT1, FOS, GSK3B, JUN, MAPK1, MAPK3, STAT1, STAT5A, IL13, IL2, IL4, and IL6) from 61 patients of the mepolizumab group who completed the MIRRA trial. The study concluded that these genetic polymorphisms did not influence mepolizumab-treatment efficacy, although this study did not check rare polymorphisms due to the small proportion of participants [25].

Although EGPA is uncommon in childhood, there has been a documented pediatric case of relapsing EGPA successfully treated with 300 mg of mepolizumab administered subcutaneously every 4 weeks. The 13-year-old girl had improvement in her respiratory and skin symptoms and she was able to reduce the intake of steroids [26]. In another case of an 18-year-old male patient with severe EGPA, the co-administration of mepolizumab and rituximab was effective in achieving remission of his symptoms in contrast to glucocorticoids and cyclophosphamide [27].

There are several studies showing the efficacy of low-dose mepolizumab (100 mg). According to the 2022 retrospective European Multicenter Study, which included 203 patients with EGPA during 2015–2020, it was explored whether a lower dose of mepolizumab (100 mg every 4 weeks) was safe and efficient for these patients. From 191 patients, 158 received 100 mg and 33 received 300 mg of mepolizumab every 4 weeks. Only 20% of the total patients were ANCA-positive, but the group who were ANCA-negative had better response rates to mepolizumab. Adverse events in the high-dose group were more common compared to the low-dose group. Both doses were almost equally effective and proved to reduce the intake of steroids and the number of eosinophils in the bloodstream [28].

Another study in 18 patients showed that 100 mg of mepolizumab was effective in controlling the symptoms of EGPA. The majority of patients (77.7%) were able to reduce their daily steroid dose and the remission rate after 12 months was 94.4%, but there was no correlation between the ANCA status and response to therapy [29]. The efficacy of 100 mg of mepolizumab has been shown in several other studies [30–33]. Mepolizumab has been successfully used in two other cases of refractory EGPA in treating pulmonary and ear lesions [34].

Ueno et al. compared the efficacy of mepolizumab in EGPA versus intravenous cyclophosphamide and found that mepolizumab was more effective in the reduction of oral steroid doses after 3 months [35]. Anti-IL5 therapy with mepolizumab in severe EGPA succeeded in the maintenance of remission, in reducing relapses, in reducing the steroid dose and in controlling active asthma in patients with severe EGPA [36, 37]. It was also found that mepolizumab may be effective in patients with peripheral neuropathy, especially with the co-administration of intravenous immunoglobulin (IVIG) [38–42].

#### Benralizumab

Benralizumab is a mAb against the IL5a receptor and it is able to induce antibody-dependent cell-mediated cytotoxicity to cells bearing this receptor, such as eosinophils. It is an antibody that has the ability to rapidly decrease the number of eosinophils both in the bloodstream and in affected tissues. Hence, it can reduce the total number of circulating eosinophils almost 100% and does not promote eosinophil degranulation, avoiding further tissue damage [43]. It is noteworthy that benralizumab has been approved since 2017 for the treatment of severe eosinophil-associated asthma [44].

So far, the largest study exploring the efficacy of benralizumab in EGPA recruited 41 patients. All patients reduced the total amount of daily prednisone below 10 mg, and 80% to less than 5 mg. Notably, 40% of the patients were able to discontinue steroids [43].

Limited evidence based on case reports suggests that benralizumab may be effective in mepolizumab-refractory EGPA [45].

According to other studies, benralizumab resulted in significant tapering and even in discontinuation of steroids especially in EGPA patients with severe asthma. The association between ANCA status and response to treatment with benralizumab remains unclear [46–48]. There is still little evidence regarding the efficacy of benralizumab in reducing ANCA titers in patients with MPO-ANCA positivity [49–51]. Moreover, benralizumab was also effective in an ANCA-negative patient with EGPA [52].

Several case reports have explored the potential role of benralizumab in EGPA [53–56]. Of interest, early administration of benralizumab in a patient with EGPA associated—biopsy proven—eosinophilic myocarditis, led to a significant increase in ejection fraction from 40 to 60%, prior to and following treatment, respectively [43, 57–59]. It has been reported that mepolizumab was unable to improve a patient with neuropathy, but when the therapy switched to benralizumab, the patient recovered [43, 57, 60]. Benralizumab was also effective in a 39-year-old patient with otitis media and sinusitis associated with EGPA [61]. As mentioned above, these are case reports, which do not prove the efficacy and safety of benralizumab in EGPA and may only trigger further research.

Finally, there is an ongoing randomized, double-blinded, active-controlled, parallel group, multicenter 52-week Phase 3 study that compares the efficacy and safety of benralizumab 30 mg versus mepolizumab 300 mg administered by subcutaneous (SC) injection in patients with relapsing or refractory EGPA on corticosteroid therapy with or without stable immunosuppressive therapy (MANDARA trial—NCT04157348).

### Reslizumab

Reslizumab is an anti-IL5 agent that has been approved by the FDA in 2016 for severe eosinophilic asthma [62]. There has been only one study reporting data regarding reslizumab in EGPA. In this pilot study, reslizumab has been administrated intravenously at a dose of 3 mg/kg in 10 EGPA patients. Reslizumab proved to be safe and effective and most of the patients achieved remission of their symptoms as well as reduction of their daily oral steroids [63]. Further and larger studies should be conducted to evaluate the efficacy of reslizumab in EGPA.

## Discussion

Recent research advances have highlighted the role of eosinophils in homeostasis and in several diseases [9]. Our knowledge of EGPA pathophysiology has expanded but the exact mechanisms are yet to be determined. Even though EGPA is classified as an ANCA-associated vasculitis, it is worthwhile exploring whether it could be also considered as an eosinophilic-driven disease. Eosinophils may drive both inflammatory and vasculitic processes and therefore may be key cells in pathogenesis. IL5 may be a significant cytokine in the pathogenesis of EGPA, by attracting and activating eosinophils in the inflamed tissues. Furthermore, some severe clinical manifestations including cardiomyopathy and neuropathy could come as a result of the overlapping influence of eosinophilic infiltration and vasculitis.

The correlation between blood and tissue eosinophilia and their clinical relevance is still unclear. Genetic factors, such as polymorphisms in the HLA-DQA1 gene, may have an impact on the prognosis and clinical outcome of the disease, but more research is needed, as mentioned above [25]. It is still uncertain whether ANCA status plays a role in anti-IL5 therapy response but some evidence points to the direction that ANCA-negative patients may have a more eosinophilic-driven pathophysiology and, therefore may better respond to anti-eosinophilic therapies. However, we should emphasize that clinical data to support this are still limited.

There is no doubt that oral steroids remain the cornerstone therapy of EGPA. Nevertheless, steroid therapy is characterized by chronic and severe adverse events and many cases of EGPA patients are steroid-refractory. Other immunosuppressive agents, such as cyclophosphamide and rituximab, have shown good results in achieving remission for a long period of time and are considered as first-line treatments, according to recent recommendations [8]. However, many patients appear to be refractory to these treatments and thus, new therapies, especially against IL5 (mepolizumab) or IL5 receptor (benralizumab) appear as attractive alternative therapeutic options. Anti-eosinophilic therapies seem to be safe and effective for the management of these patients by reducing clinical manifestations in active and severe EGPA, inducing remission and minimizing the daily administration of oral steroids. Mepolizumab and benralizumab have also shown efficacy in patients with refractory EGPA to other medications. The fact that many patients do not respond to anti-IL5 treatment may suggest that there are more complex inflammatory pathways and targeting only one mechanism may not be enough.

In conclusion, anti-eosinophilic therapies appear as attractive therapeutic options for patients with EGPA with a good efficacy and safety profile. For patients with severe disease, current recommendations indicate steroids alongside RTX or CYC as first-line treatments. Data regarding anti-eosinophilic therapies in patients with severe disease are limited since these patients are usually excluded from clinical trials. However, in patients with active, non-severe disease, steroids and mepolizumab are recommended as first-line treatments. It is noteworthy that anti-eosinophilic therapies have shown efficacy in patients with severe disease refractory to standard therapy and as our clinical experience with the use of these agents is expanding, their place in the treatment algorithm may change. For the time being, we have three main therapeutic options namely, CYC, RTX and anti-eosinophilic therapies apart from steroids which are used universally in all patients. Up until now, we lack reliable biomarkers that could predict response to treatment and this should be an area of research in the near future. We certainly need biomarkers that could pinpoint patients with a prominent vasculitic pathophysiology versus patients with a predominant eosinophilic-driven disease; the former patients may respond better to RTX or CYC whereas the latter may respond better to anti-eosinophilic therapies. Taking into account that vasculitis and eosinophilic-driven pathology overlap in patients with EGPA, combination treatment targeting both pathogenetic arms could also be explored in patients with severe/refractory disease.

## Data Availability

All data are presented in the current manuscript.
